# Interference of Biodegradable Plastics in the Polypropylene Recycling Process

**DOI:** 10.3390/ma11101886

**Published:** 2018-10-02

**Authors:** María Dolores Samper, David Bertomeu, Marina Patricia Arrieta, José Miguel Ferri, Juan López-Martínez

**Affiliations:** 1 Instituto de Tecnología de Materiales, Univesitat Politècnica de València, 03801 Alcoy-Alicante, Spain; daberpe@alumni.upv.es (D.B.); joferaz@upvnet.upv.es (J.M.F.); jlopezm@mcm.upv.es (J.L.-M.); 2 Instituto de Ciencia y Tecnología de Polímeros (ICTP-CSIC), 28006 Madrid, Spain; marina.arrieta@gmail.com

**Keywords:** recycling, polypropylene, biodegradable polymers, degradation, inmiscibility

## Abstract

Recycling polymers is common due to the need to reduce the environmental impact of these materials. Polypropylene (PP) is one of the polymers called ‘commodities polymers’ and it is commonly used in a wide variety of short-term applications such as food packaging and agricultural products. That is why a large amount of PP residues that can be recycled are generated every year. However, the current increasing introduction of biodegradable polymers in the food packaging industry can negatively affect the properties of recycled PP if those kinds of plastics are disposed with traditional plastics. For this reason, the influence that generates small amounts of biodegradable polymers such as polylactic acid (PLA), polyhydroxybutyrate (PHB) and thermoplastic starch (TPS) in the recycled PP were analyzed in this work. Thus, recycled PP was blended with biodegradables polymers by melt extrusion followed by injection moulding process to simulate the industrial conditions. Then, the obtained materials were evaluated by studding the changes on the thermal and mechanical performance. The results revealed that the vicat softening temperature is negatively affected by the presence of biodegradable polymers in recycled PP. Meanwhile, the melt flow index was negatively affected for PLA and PHB added blends. The mechanical properties were affected when more than 5 wt.% of biodegradable polymers were present. Moreover, structural changes were detected when biodegradable polymers were added to the recycled PP by means of FTIR, because of the characteristic bands of the carbonyl group (between the band 1700–1800 cm^−1^) appeared due to the presence of PLA, PHB or TPS. Thus, low amounts (lower than 5 wt.%) of biodegradable polymers can be introduced in the recycled PP process without affecting the overall performance of the final material intended for several applications, such as food packaging, agricultural films for farming and crop protection.

## 1. Introduction

The world plastic production has reached more than 330 million tons in the last few years. Among all plastics, polypropylene (PP) is the most demanded for plastic converter industries in Europe [1]. In fact, PP is one of the most used and consumed polymers in the world due to its good processing performance and versatility; it is used for a wide variety of applications: commodities, medical applications, automotive, etc. It is known as one of the “packaging plastics” and packaging products are mainly short-term applications which ultimately represent a big source of plastic waste. Thus, a large amount of PP waste is produced every year after its useful life. Fortunately, a huge part of plastic residues (more than 30%) are retrieved using industrial recycling, closing the loop of circular economy [1]. Particularly, recycled PP can be used in different ways like new packaging products, films or matrix of wood composites [2,3]. Moreover, recycled PP can be considered a safe material because the producers do not usually use hazardous materials in its process. However, we must take into account that some recycled materials can be hazardous such as granulated end-of-life tyres because they can contain polycyclic aromatic hydrocarbons (PAHs), some of which are identified as carcinogens. Also, recycled expended polystyrene (EPS) coming from building and construction sector can be considered a hazardous waste because EPS is highly combustible and flame retardant hexabromocyclododecane (HBCDD). It had been frequently added until it was included in the reach regulation list in 2015 because it is considered a persistent Organic Pollutant (POPs) [4]. The best option for disposal hazardous polymers waste is energy recovery, since it can meet partial energy demand and reduce disposal cost, including CO_2_ emissions [5].

Another interesting approach to close the loop of circular economy is the use of biobased and biodegradable polymers, known as biopolymers, which have non-dependence on petrochemical resources and also do not represent an environmental potential hazard if they ultimately reach landfills. Therefore, in recent years a great interest on the use of biobased and biodegradable polymers has increased in order to replace the petrochemicals-based packaging materials and to reduce plastic waste in landfills in certain applications, mainly short-term packaging and agricultural films [6,7,8]. Thus, the use of biodegradable plastics is rising, mainly because there is an increasing concern about the reduction of the plastics’ environmental impact. In fact, currently, industries and consumers demand these types of products on the market. According to Bastioli et al., Europe should take advantage of the great potential of these materials to add value to products by taking advantage of the new bio-economic feature of bioplastics, as well as to preserve and improve ecosystems and biodiversity [9,10].

Biopolymers [11], such as poly (lactic acid) (PLA) [12,13], polyhydroxybutyrate (PHB) [14] and thermoplastic starch (TPS) [15], are increasingly used in the food packaging and agricultural sector, in addition to other fields of application such as medical [16,17,18] or composite materials [18,19,20]. However, consumers have low information about where they have to throw away this kind of plastics after their useful life and they are commonly disposed of with traditional waste plastics [21]. Although the bioplastic products can also be recycled after their use by recycling in traditional ways [22], the current systems do not allow the recovery of high purity of plastic waste. Moreover, these new technologies increase the cost of the final product developed with recycled biopolymers. On the other hand, if biodegradable packaging residues are found in recycling channels, they could act as impurities for traditional plastics influencing the structural and thermal properties of recycled products. The separation and classification processes of these biodegradable products can be complex and expensive [23,24,25] and if the consumption of bio-based plastics continues to increase, as it has been predicted, current recycling systems will have to be considered a reorganization to avoid contamination of recycled plastics [26]. Currently, there have not been found in the literature works on the mixture of small % of biopolymers in a PP matrix that help to evaluate their inclusion in the recycling of this material. However, different studies of PP/bioplastics blends have been carried out using different compatibilizing agents. This is the case of PLA/PP blends, where studies have been conducted with different amounts of compatibilizing agents such as polypropylene-graft-maleic anhydride (PP-g-MAH) and styrene-ethylene-butylene-styrene-graft-maleic anhydride (SEBS-g-MAH), these compatibilizers improve some properties such as impact strength, especially using a 3 phr of PP-g-MAH [27,28,29]. Studies have also been carried out on PP/TPS blends with different percentages of TPS, observing that the increase in TPS causes a decrease in tensile strength, elongation at break and MFI [30,31]. In the case of PP/PHB mixtures, not too much information was found, Sadi et al. performed a work on the compatibility of PP/PHB blends with 20 wt.% of PHB using different compatibilizers, the PHB causes a significant decrease in the mechanical properties of PP that can be improved using poly (ethylene-co-methyl acrylate-co-glycidyl methacrylate) (P(E-MA-GMA)) [32].

It is expected that in the near future, the consumption of biodegradable polymers will grow up and the conventional recycled polymers may have a low amount of biodegradable polymers acting as impurities. The presence of small fractions of impurities can negatively influence the structural and mechanical properties of the conventional recycled materials, which might decrease their price and viability [33]. This lack of properties is due to the incompatibility of the polymeric components in the blend. In fact, blending approaches use a number of compatibilization strategies that in general are related to the addition of a third component that is miscible with both phases (i.e., co-solvent, nanoparticles), or one part of the third component that is miscible with one phase and another part with another phase (i.e., copolymers) [34,35]. Compatibilizers can be used to balance not only the loss of mechanical properties but also the morphological changes of the immiscibility of polymers, as suggested by MacAubas et al. and Fekete et al. [36,37]. However, considering the industrial plastic recycling process, it is expected that small fractions of impurities reach the PP recycling process without any kind of compatibilizers.

In this work, blends based on recycled PP and the most typically used biodegradable polymers in short-term applications were studied in order to simulate recycled PP contaminated with low amounts of PLA, TPS and PHB up to 15 wt.%. With this purpose, five different percentages of biodegradable plastics were blended with polypropylene and further processed by melt extrusion followed by an injection molding process to simulate the most typically used processing technologies at industrial level. Then, the effect of the biodegradable materials presence on the mechanical and thermal properties were evaluated. Therefore, the changes on the softening temperature (VICAT) and the melt flow index were studied. The mechanical properties were also analyzed to determine the influence of the biodegradable plastic presence on the extruded blends on the mechanical performance of the final materials. Furthermore, FTIR studies were carried out to easily determine the presence of biodegradable materials in recycled polypropylene, while scanning electron microscopy (SEM) was used to evaluate the polymer-polymer microstructural interaction. Additionally, since a huge amount of plastic still ends in landfill the blends were also exposed to composting conditions at a laboratory scale level in order to get information about the influence of biopolymers into recycled PP under environmental composting conditions. The results allowed to identify the maximum amount of biodegradable materials that can be blended with recycled PP as impurity without compromising the mechanical and thermal integrity of the PP based products.

## 2. Materials and Methods

### 2.1. Materials and Preparation of the Blends

Recycled PP, with reference PP1B, has been supplied by Acteco (Ibi, Spain), PLA 4032D by NatureWorks LLC (Minnetonka, MN, USA), TPS Mater Bi by Novamont (Novara, Italy) and PHB P226 by Biomer (Krailling, Germany).

The blends were made by mixing PP with different percentages of biodegradable polymers, that ranged from 0 to 15 wt.%, as can be seen in the Table 1, in a twin screw extruder (Dupra S.L., Castalla, Spain), processed at a temperature range of 200–220 °C at 50 rpm. The blend samples were then injected by an injection molding process using a Babyplast estandar 6.6 (Cronoplast S.L., Albrera, Spain) machine with a mold with normalized sample dimensions for tensile test according to ISO-527-2, specifically 5A samples.

### 2.2. Scanning Electron Microscopy Analysis

The Scanning Electron Microscopy (SEM) images were took with a Phenon of FEI equipment (Eindhoven, The Netherlands) using 5 kV voltage, to observe the miscibility of the components in the blends subjected to a cryofracture process. Before the observation, the samples were coated with a gold-palladium alloy by a Sputter Mod Coater Emitech SC7620 (Quórum Technologies, East Sussex, UK).

### 2.3. Infrared Spectroscopy Analysis

The infrared spectroscopy analysis was conducted using a Perkin Elmer Spectrum BX spectrometer (Perkin-Elmer España S.L., Madrid, Spain). The test was made with 20 scans between 600 and 4000 cm^−1^ with a resolution of 32 cm^−1^ mode using an attenuated total reflectance (ATR) accessory, indicated for samples with poor transparency.

### 2.4. Thermal Characterization

#### 2.4.1. Thermogravimetric Analysis (TGA)

Dynamic thermal degradation analysis was carried out using thermogravimetric analyzer TGA/SDTA 851 Mettler Toledo (Schwarzenbach, Switzerland). TGA measurements were run at 20 °C·min^−1^ constant heating rates. Temperature was raised from 30 to 600 °C under air conditions in order to study oxidative degradation process following the conditions used in a previous work [38]. The initial degradation temperature (T_0_) was calculated at 5% mass loss, while temperatures at the maximum degradation rate (T_max_) for each stage were determined from the first derivatives of the TGA curves (DTG).

#### 2.4.2. Differential Scanning Calorimetry

The differential scanning calorimetry (DSC) was conducted with a Mettler Toledo 821 equipment (Mettler Toledo, Schwerzenbach, Switzerland) using samples of 4–6 mg. The heating and cooling programs were performed at a 20 °C·min^−1^ speed in a nitrogen atmosphere (60 mL·min^−1^). The DSC program was carried out in three stages: the first heating took place from 30 to 200 °C, followed by a cooling process up to 30 °C to −20 °C·min^−1^ followed by a second heating up to 250 °C. The first heating was carried out to remove the thermal history of the materials. The melting temperature, T_m_, and the melting enthalpy, ΔH_m_, were obtained from the second heating.

### 2.5. Mechanical Properties

The tensile test properties were performed with a universal testing machine Ibertest ELIB 30 (SAE Ibertest, Madrid, Spain) at room temperature, according to ISO 527; the tests were performed with a load cell of 5 kN and at a speed of 10 mm·min^−1^. From each sample type at least 5 specimens were tested and the mean of those tests was calculated.

The Shore D hardness was measured according to the UNE-EN ISO 868 standard using a hardness equipment Mod.673-D (Instruments J. Bot S.A., Barcelona, Spain). The results were the mean hardness of at least 5 measurements of samples with thickness of 4 mm.

### 2.6. Exposition to Composting Medium

The PP based blends were exposed to compost condition with the main objective to study the influence of PLA, PHB and TPS into PP based blends disintegration. The disintegration under composting conditions was performed at laboratory scale level according to the ISO 20200 standard [39]. Dogbone samples were buried at 4–6 cm depth in perforated plastic boxes containing a solid synthetic wet waste (10% of compost (Mantillo, Spain), 30% rabbit food, 10% starch, 5% sugar, 1% urea, 4% corn oil and 40% sawdust as well as approximately 50 wt.% of water content) and were incubated at aerobic conditions (58 ± 2 °C). PP based blends were recovered at 8, 21 and 30 days. A qualitative check of the physical disintegration in compost as a function of time was done by taken photographs, while the structural changes were followed by TGA measurements conducted from 30 to 600 °C at 20 °C·min^−1^ under oxidation conditions.

### 2.7. Other Techniques

The VICAT (VST) softening temperature was studied with the VICAT/HDT station DEFLEX 687-A2 (Metrotec S.A., San Sebastián, Spain) according to the ISO 306, to 50 N with a heating rate of 50 °C·h^−1^. The flow index measures of the different blends were performed according to ISO113, using 2.16 kg and 230 °C, with an extrusion plastometer (AtsFaarS.p.A., Vignate, Italy).

## 3. Results and Discussion

### 3.1. Miscibility

The miscibility between different polymers depends on the chemical structure of the polymers as well as on their crystalline nature and morphology of the starting polymers [23,40]. While miscibility is limited to a specific set of conditions, several polymers form immiscible blends. The incompatibility between two polymeric matrices causes the loss of the mechanical properties and even superficial lamination. This loss of properties also depends on the percentages of each component on the blend sample. In the present work, it seems that biodegradable polymers are acting as impurities, probably due to their different polarities. It is known that the relative affinity between two polymers can be estimated using the solubility parameter (*δ*) [41]. To consider that a mixture’s components are compatible, their solubility parameter should be similar. In this sense, δ should be calculated taking into account the contribution that each group has in the overall structure of the molecule (Equation (1)).

(1) $$\delta =\frac{\rho \Sigma_{j}F_{j}}{M_{n}}$$

where *δ* ((cal·cm^−3^)^1/2^) is the solubility parameter for each component, *ρ* (g·cm^−3^) is the polymer density, *Mn* (g·mol^−1^) is the molar mass of the repeated unit, *∑_j_* and *F_j_* are the sum of the contributions of all groups (*F*, (cal·cm^−3^)^1/2^·mol^−1^).

**Figure 1:**
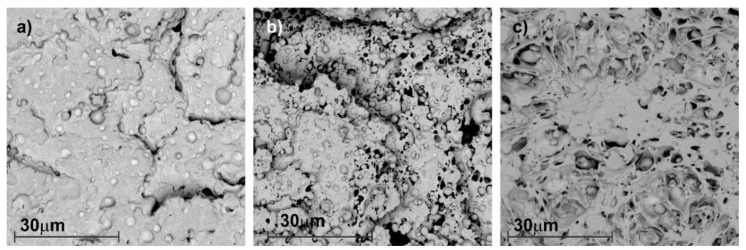
SEM Images at 3500× magnification of the samples. (**a**) PP-15PHB; (**b**) PP-15PLA; (**c**) PP-15TPS.

The results of the calculated *δ* can be seen in Table 2, where *δ* was calculated according to the Small method, using Equation (1) and the values of F of Table 3, the solubility parameter results were very similar with available data in polymerdatabase.com for the polymers studied [42]. While the *δ* of PP is 16.4 MPa^1/2^, that of PLA is 19.5 MPa^1/2^ and it is the biodegradable polymer with the nearest *δ*. Although the solubility values of these two polymers are close, it is not enough to consider these two materials miscible. Regarding the biodegradable polymers TPS and PHB, whose solubility parameter are 8.4 MPa^1/2^ and 21.4 MPa^1/2^, respectively. Thus, they clearly indicate that there is an increased immiscibility with the PP matrix, since the solubility parameter of these polymers is more distant from PP. Therefore, according with the solubility parameter results it seems that the biodegradable materials studied here are not miscible with PP and, for that reason, they could generate a thermal and mechanical properties deterioration on the recycled PP. The microstructural analysis was performed by SEM. In Figure 1, we observed the SEM images of the different PP blends with 15 wt.% of different biodegradable polymers as an example, PLA (Figure 1b), PHB (Figure 1a) and TPS (Figure 1c). It can be clearly seen that the blends based on PP and biodegradable polymers studied here are immiscible, since a phase separation of the components in the different blends can be observed. In fact, all blends samples exhibit spherical droplets dispersed in the PP matrix. Some of the spherical droplets have been pulled out of the PP matrix during fracture, indicating very weak interfacial adhesion and immiscibility between both polymers, particularly in the case of PP-TPS blend which showed higher spherical droplets (Figure 1c). This could be due to the polyolefin structural differences in comparison with the biodegradable polymers, as predicted using the solubility parameter. When two polymers are immiscible and are blended together, a two-phase system is formed. Generally this material has low mechanical properties due to the stress concentration generated by the poor adhesion between the phases [43,44].

### 3.2. Detection of Biodegradable Materials in the Recycled PP Using the FTIR Technique

**Figure 2:**
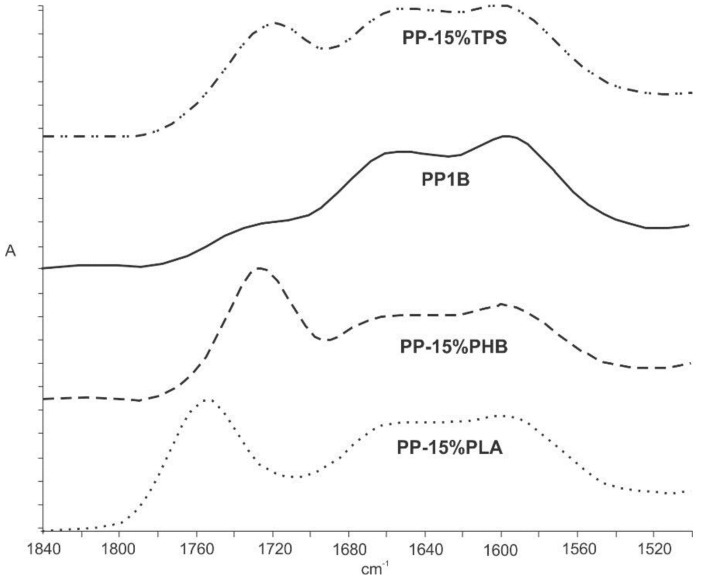
FTIR spectra: PP (PP1B) and PP with 15% biodegradable polymer.

Through the FTIR technique, biodegradable materials can be easily detected in the recycled PP, since, as it was demonstrated in our previous work, some of the characteristic bands of the biodegradable polymers (PLA, PHB and TPS) do not overlap with the PP characteristic bands [38]. As shown in Figure 2 between 1700 and 1800 cm^−1^, the PP/biodegradable polymers blends (with 15 wt.% of the different biodegradable polymers) exhibit a strong band that has no presented the neat recycled PP. This is due to PLA and PHB present the carbonyl group (–C=O) characteristic band at this wavelength. The asymmetric stretching of the carbonyl group in neat PLA is at higher wavelength (1754 cm^−1^) and it is attributed to the amorphous carbonyl vibration. Meanwhile, the stretching vibration of crystalline carbonyl groups is centered at lower wavelengths (1726 cm^−1^) in the spectrum of neat PP-15%PHB associated with the crystalline state of PHB [45]. TPS presents the same band due to the additives used for their manufacturing in the thermoplastic form [38]. Although FTIR technique does not allow to quantify the amount of biopolymer in the blends, it represents a simple and fast method to detect the presence of this kind of impurities in the recycled PP process which is easily scalable up to the plastic recycling industry.

### 3.3. Thermal Characterization

#### 3.3.1. Thermogravimetric Analysis

**Figure 3:**
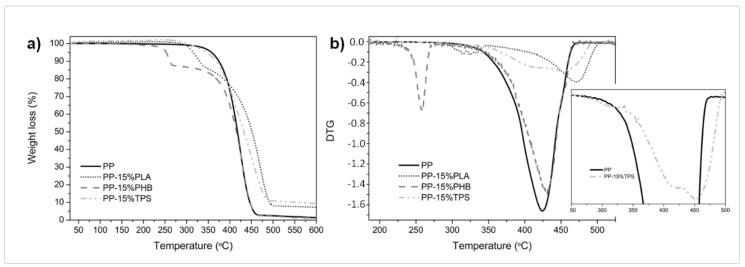
(**a**) TGA and (**b**) DTG thermograms of PP blends with 15 wt.% of biodegradable polymers.

Since the amount of different components in a polymeric blend sometimes can be estimated from TGA, the thermal decomposition of the blends was studied by means of TGA and DTG. In Figure 3, we show the TGA (Figure 3a) and DTG (Figure 3b) results of PP blends blended with 15 wt.% of biopolymers as example. Moreover, the thermal degradation is very important for the plastic processing industry since biopolyesters thermal degradation could lead to the formation of oligomers, such as oligomeric lactic acid (OLA) in the case of PLA and oligomers of 3-hydroxybutyrate (OHB) in the case of PHB, which can further act as plasticizers. TGA shows a complete weight loss of PP in a single degradation step. Meanwhile, PP blended with PLA and PHB were degraded in two steps, where the first one is assigned to the biopolyesters decomposition and the second one, at higher temperatures, was related to the PP thermal degradation. TGA revealed that all PP based blends showed minor thermal stability with respect to PP sample (PP T_0_ = 357 °C). TPS was the biopolymer that less shifted the onset degradation temperature to lower values, around 10 °C for PP-15%TPS, T_0_ = 346 °C. Higher reduction in onset thermal degradation was observed when biopolyesters were blended with PP, particularly in the case of PHB (PP-15%PLA T_0_ = 315 °C and PP-15%PHB T_0_ = 315 °C). Nevertheless, it should be highlighted that no degradation takes place in the temperature region from room temperature to 220 °C, which is the temperature range where the blend samples were processed. While FTIR allowed to identify the presence of biopolymers in the recycled PP, TGA allows to estimate the amount of biopolyesters in the blends. For instance, from Figure 3a, the loss of both biopolyesters could be estimated from TGA and, as it is expected, it is around 15%. This result confirms that there were not thermal degradation of biopolyesters during processing, and it is particularly important for PP-PHB blends since it is known that the foremost drawback for the industrial production of PHB based blends is its small processing window [21]. Different situation is observed for PP-TPS based blends, since the degradation take place in one step process like PP, avoiding the possibility to quantify the amount of TPS as impurities in the blend. Although the amount of TPS could not be quantified, the contamination of PP could be identified from DTG curve (Figure 3b) in which is possible to observe that the degradation starts prior to the degradation process of PP (see the shoulder in the insert Figure 3b), which has been attributed to the starch pyrolysis (between 300 and 360 °C) [46]. In addition, after the main degradation process of PP there is another degradation step between 360 and 500 °C that has been related to the oxidation of the partially decomposed starch in air atmosphere [47] and to the decomposition of the biodegradable co-polyester component in TPS [46]. Moreover, the maximum degradation temperature of PP was shifted from 423 °C in PP to 456 °C in PP-15%TPS suggesting somewhat positive interface interaction between PP and biodegradable TPS material. Biopolyesters also shifted the maximum degradation temperature of PP to higher values (T_max_PP in PP-15%PLA 474 °C and in PP-15%PHB 430 °C).

#### 3.3.2. Differential Scanning Calorimetry

**Figure 4:**
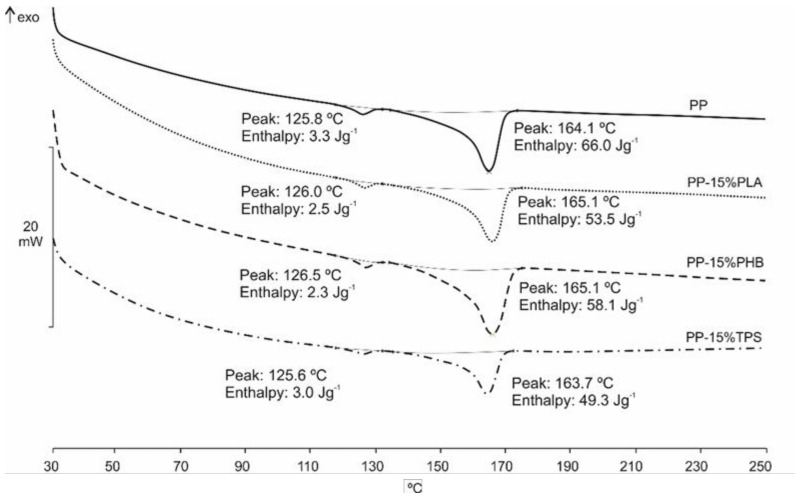
DSC curves of PP blends with 15 wt % of biodegradable polymers.

**Figure 5:**
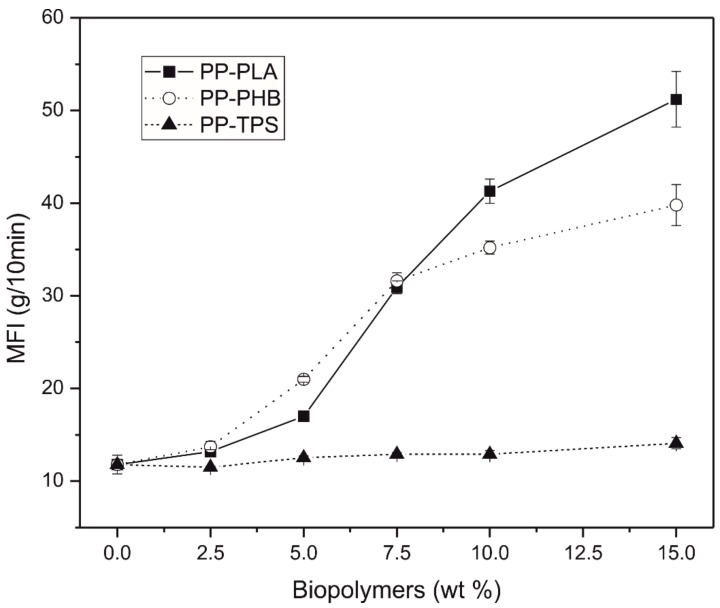
Plot of MFI vs. wt.% biodegradable polymer.

In Table 4 and Figure 4, we show the effect of the presence of different biodegradable polymers on the thermal properties of the recycled PP measured by DSC. In Figure 5, we show the second heating of the samples with 15% of bio-based polymers, the recycled PP calorimetric curve had 2 melting peaks, the second correspond with melting peak of PP and the little first peak could be a contamination with another polymer like a HDPE or LDPE. This double melting peak can be also observed in the other PP with bio-based DSC curves. In Table 4 can be observed that the melting temperature, T_m_, does not vary and it is between 163.4 and 165.1 °C. Compared to the T_c_, obtained from the DSC cooling process, it can be observed that PP presents a crystallization temperature at 124.5 °C and in all samples containing biodegradable polymers, either PLA, PHB or TPS, the crystallization temperature decreased, being between 120.5 and 121.6 °C. In general, the presence of biodegradable polymers in the PP matrix caused a decrease of the crystallinity, as the enthalpy values of crystallization and melting decreased. The decrease of crystallinity may be due to the fact that biodegradable polymers in the blend make difficult the pack of PP chains, since the presence of biodegradable polymers acts as impurities, and thus, they would reduce the free volume of PP [48,49].

Therefore, the presence of biodegradable polymers not only affects the mechanical properties of the recycled PP as it will be discussed in the following sections, but also affects the thermal performance, especially the PP crystallinity considering that the melting temperature is only slightly modified.

### 3.4. Thermomechanical Characterization

Previously, we discussed the changes caused by the presence of biodegradable polymers on the thermal properties of the different blends studied but not only these properties are important in the polymeric materials recycling. Therefore, the changes on thermomechanical properties were also taken into account, as they are too important mainly for the polymer processing industry.

Figure 5 shows the graphical representation of the melt flow index (MFI). It can be seen that the TPS does not significantly modify the MFI of the PP, since it practically remains constant for all the percentages studied, despite being the polymer with the farthest solubility value compared to PP. In the case of blends made with PP with PLA or PHB, it is observed that the MFI increases as the percentage of biodegradable polymer in the blends increase. This increase is more pronounced for the samples made with PLA. Nevertheless, blends prepared with low amount (2.5 wt.%) of these biodegradable polymers, that is PP-2.5%PLA and PP-2.5%PHB, MFI is not practically affected. This could be related with the fact that increasing the polyester amount in the blend, the amount of ester groups, which are relatively easy to breakdown and have poor thermal stability [50], increases and more chain scission occurs leading to an increase in MFI.

**Figure 6:**
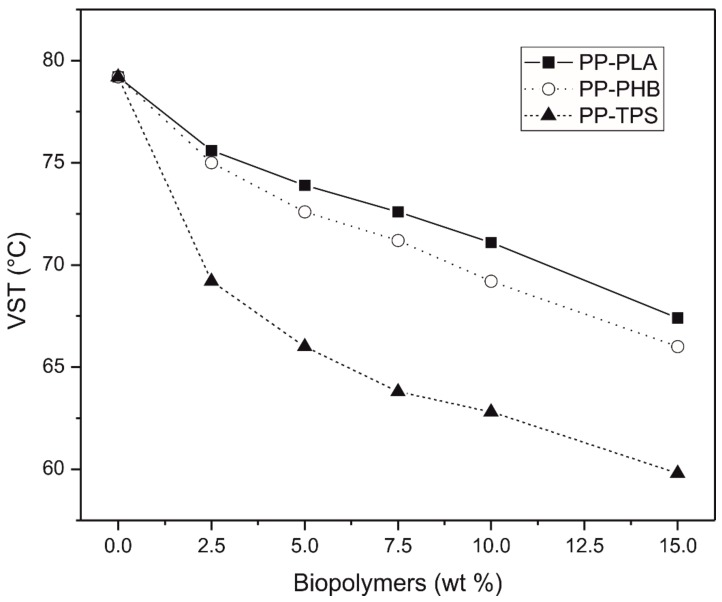
VST vs. wt.% biodegradable polymer.

The blend thermal stability was studied by determining the softening temperature VICAT (VST). The results for all the studied systems, PP-PLA, PP-PHB and PP-TPS, show the same behavior, since as the amount of biodegradable polymer increases it causes a decreases of the VST (Figure 6). Depending on the system, the decrease of this property is more or less pronounced. While the PP-PLA blend system is the one with the highest VST (lower VST reduction), TPS is the biodegradable material that causes a greater decrease of this property. This behavior could be related to the fact that starchy materials are water sensitive and are able to show a rubber-like behavior depending on its moisture content [51].

### 3.5. Mechanical Characterization

**Figure 7:**
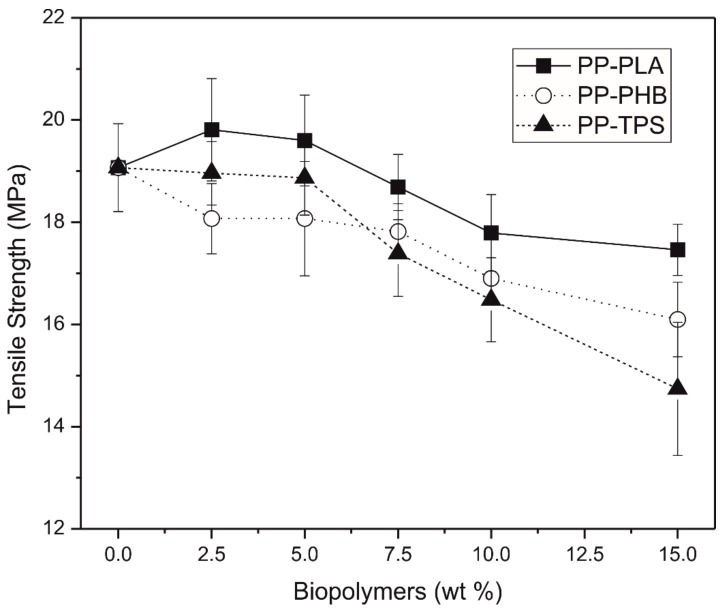
Variation of tensile strength vs. wt.% biodegradable polymer in polypropylene.

**Figure 8:**
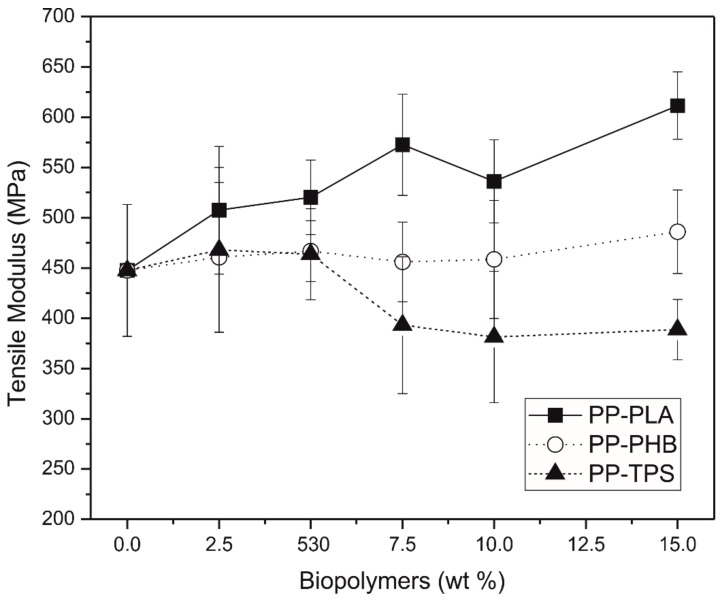
Variation of modulus of elasticity vs. wt.% biodegradable polymer in polypropylene.

The determination of the mechanical properties in blends is very important because of the incompatibility of different polymers negatively affects the material performance, causing a decrease on the mechanical properties [36]. A small alteration can be observed on tensile strength (Figure 7) and tensile modulus (Figure 8) due to the different biodegradable polymers presence, while these differences increased as the biodegradable polymers percentage increased up to 5 wt.%.

Figure 7 shows the variation in the tensile strength of the different blends made with PP and biodegradable polymers. It is observed that percentages lower than 5% of PLA and TPS do not significantly vary the tensile strength of the recycled PP, since it practically remains constant. However, larger quantities of these two polymers decrease the tensile strength. On the other hand, blends made with PP-PHB show a decrease in tensile strength in all percentages. This decrease in strength is due to the lack of interaction between the polymers blends at the interfaces. With regard to the elongation at break, the results showed (Table 5) that this property not change significantly with the addition of different bioplastics studied, maybe due to the recycled PP used in this work has a very low level of elongation. Although these results show scattered values, it seems that biopolymers are not acting as plasticizer for the PP matrix, in good agreement with thermal degradation results in which it was observed that there were not thermal degradation of biopolyesters during processing, which would lead to the formation of oligomers able to plasticize the polymeric matrix.

The graphical representation of the tensile modulus (Figure 8) shows that the variation of this property depends on the biodegradable polymer used in the blend. In blends made with PP-TPS it is possible to observe that the elastic modulus remains constant when the amounts of TPS added are lower than 5 wt.%. For higher values it can be seen that the modulus decrease considerably up to 400 MPa. In the PP-PHB blends the modulus remains practically constant around 450 MPa for all the percentages studied, this may be because the PHB has an elastic modulus similar to that of the PP [52]. On the other side, in PP-PLA blends, it can be observed that the elastic modulus increases as the PLA content increases from around 450 for recycled PP to around 610 MPa for PP-15% PLA, this increase may be due to the fact that PLA possess a higher elastic modulus than PP [53].

The results obtained in the mechanical characterization were in good accordance with the calculated solubility parameters. PLA is a biodegradable polymer with the closest δ to that of the PP and the presence of low percentages of this polymer less or equal to 5 wt.% of PLA, did not cause a decrease on the mechanical properties, but in higher percentages the tensile strength and the tensile modulus decreased. Similar findings were found in the study carried out by Pivsa-Art et al. who analyze PLA-PP blends, the incorporation of 20 wt.% of PLA into PP matrix caused a slight increase in tensile strength and Young’s modulus. However, to improve the mechanical properties of PLA-PP blends they used polypropylene grafted with maleic anhydride as compatibilizer [27]. Regarding the PHB and TPS presence in the recycled PP, they caused the mechanical properties deterioration. For instance, it happened to Sadi et al. who performed a study on PP blends with 20 wt.% of PHB. The mechanical properties of this blend were lower than that of PP, and thus, they studied the compatibilization with different copolymers, founding that the most effective approach was using poly (ethylene-co-methyl acrylate-co-glycidyl methacrylate) [32].

In a study conducted by Kaseem et al. on blends made with PP and TPS, the increase of TPS caused a decrease on the tensile strength since the immiscible TPS acted as a filler for PP matrix [30].

### 3.6. Desintegration under Composting Medium

**Figure 9:**
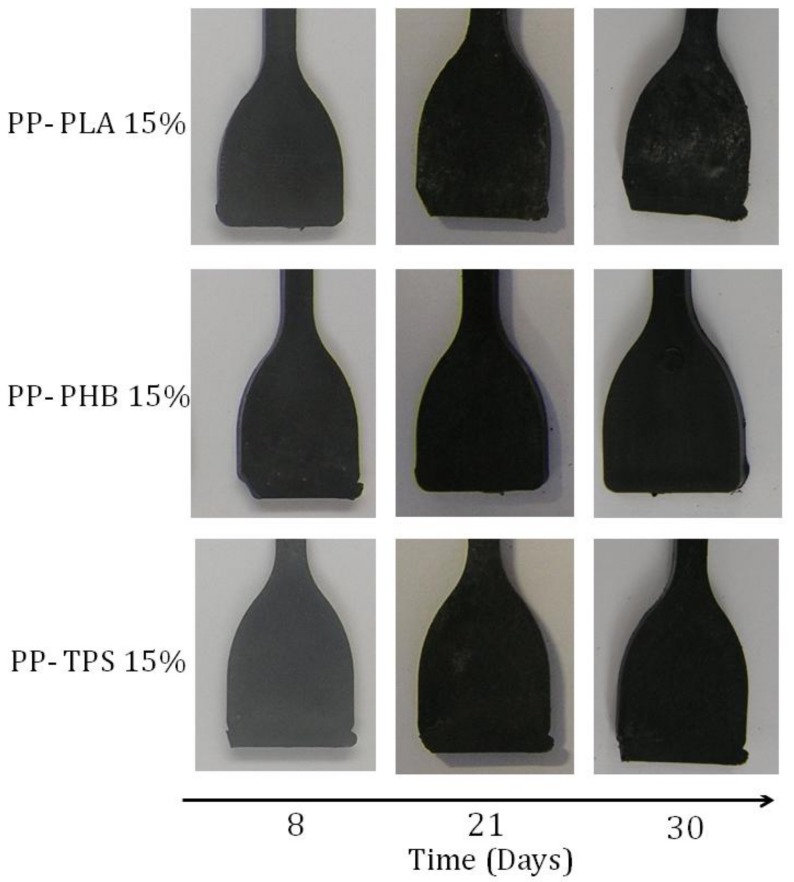
Visual appearance of recovered blend samples at different times of composting (8, 21 and 30 days).

**Figure 10:**
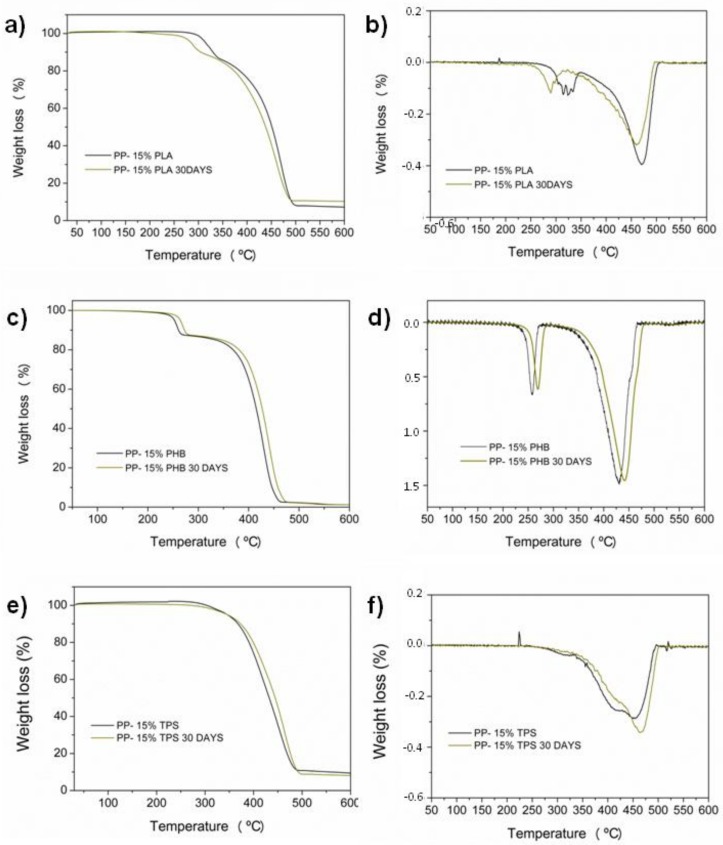
TGA (**a**,**c**,**e**) and DTG (**b**,**d**,**f**) thermograms of PP- biodegradable blends before and after 30 days exposed to a composting medium.

Unfortunately, instead of reaching the recycling system, several PP-based products still go to landfill after their useful life, and thus in order to simulate this end of life option the materials were exposed to composting conditions at laboratory scale level. It is known that PLA, PHB or starch-based materials are totally disintegrated under composting medium exposed to thermophilic aerobic conditions [21,47], that is according to the ISO standard in less than three months [39]. In fact, blends containing PLA, PHB or TPS in their formulations requires between one and two months to be completely disintegrated under composting [54,55]. Meanwhile, since PP is not a biodegradable polymer it is not suitable to perform disintegration in a composting medium. Thus, the PP blends were subjected under controlled composting conditions during 1 month. The visual appearance of recovered samples at different time of exposition in composting conditions (8, 21 and 30 days, on the basis of previous work [38]) are shown in Figure 8. It was observed that the samples suffered somewhat physical changes at the surface after 21 days and mainly after 30 days, suggesting that the biodegradation of PLA, PHB and TPS is taking place. Therefore, TGA analysis were conducted to follow the loss of biodegradable materials during composting exposition as it was previously reported for polystyrene/biopolymers blends [38]. In Figure 9 are shown the TGA and DTG curves of PP-15%PLA (Figure 10a,b), PP-15%PHB (Figure 10c,d) and PP-15%TPS (Figure 10e,f) before and after 30 days exposed to composting conditions. As it was already commented in TGA results, in the case of PP-15%PLA and PP-15%PHB blends the thermal degradation takes place in two-step process in which the first step is related with the loss of the biodegradable material, PLA or PHB, and the second one corresponds to the degradation of the PP. After 30 days in composting the onset degradation temperature of PP-15%PLA blend was considerably reduced (Figure 10a), since the disintegration of PLA is taking place and thus there are shorter PLA chains, such as oligomers, which present lower thermal stability [13], which degrade faster than longer PLA polymeric chains. Similarly, the maximum degradation temperature corresponding to PLA (T_max_PLA) at about 325 °C was shifted to 289 °C after 30 days (Figure 10b). The second maximum degradation temperature of PP-15%PLA was about 472 °C before composting, while after 30 days in composting it was shifted towards lower temperatures (462 °C) approaching to that of PP because there is less PLA impurities in PP matrix at this composting stage. In the case of PP-15%PHB (Figure 10c) the onset degradation temperature was shifted to higher temperatures, since PHB has lower thermal stability than PP. The maximum degradation temperature of PHB (T_max_PHB = 257 °C) was shifted to higher values during composting reaching 270 °C after 30 days (Figure 10d), because of the blend behaves more similarly to PP while it loss the PHB. Similarly, the second maximum degradation was shifted from 430 °C to 442 °C during composting. Finally, for PP-15%TPS the thermal degradation take place in only one step and the onset degradation temperature was around 346 °C in PP-15%TPS blends (Figure 10e) and this value was maintained after 30 days in composting. Nevertheless, the shoulder observed just before the maximum degradation temperature corresponds to the cleavage of ether linkages in starch backbone of TPS [47] and it was slightly reduced (Figure 10f). Meanwhile, there was a second peak at higher temperatures, at about 450 °C in Figure 10f, which was shifted to 465 °C after 30 days in composting. The displacements of the maximum degradation temperatures were more pronounced in the case of PP-PLA and PP-TPS blends, since there were less amount of biodegradable material in both formulations after the exposition to the composting medium. Meanwhile, the more crystalline PHB was less disintegrated at this stage of disintegration. However, it should be mentioned that in all cases still remains biodegradable polymer in the formulations, showing that PP can limit the exposure of biodegradable polymers to the composting degradation suggesting that there is somewhat positive interface interaction between PP and biodegradable materials as it was observed in TGA result (Section 3.3.1) and/or the blend separation is forcing a discontinuous disintegration, and thus, biodegradable materials are less available for the hydrolysis and the further microorganisms attack in the composting medium.

## 4. Conclusions

In this study, the microstructural, thermal and mechanical properties of blends based on recycled PP with different biodegradable polymers (PLA, PHB and TPS) as impurities were evaluated. The presence of biodegradable polymers in recycled PP caused a significant loss of mechanical, thermomechanical as well as thermal properties, especially when using percentages of biodegradable polymers higher than 5 wt.%. In addition, the effect of the presence of the biodegradable polymers resulted in evident features seen in SEM images, where the immiscibility of the blends was clearly observed by the presence of two separated phases. The exposition of PP-based blends to composting medium showed that although the PP-biodegradable polymer blends were mainly immiscible, they had somewhat positive interactions with PP matrix, since biodegradable polymers delay their disintegration process. As the thermal and mechanical properties of the recycled PP are affected by the presence of more than 5wt.% of PLA, PHB and TPS biodegradable polymers, it is very important to be able to detect biodegradable materials in PP recycling process. The FTIR technique allowed to easily detect the presence of biodegradable polymers in the recycled PP by the appearance of the –C=O characteristic band of PLA, PHB and TPS between 1700–1800 cm^−1^. Meanwhile, TGA results and effective technique to quantify the presence of biopolyesters PLA and PHB in the recycled PP. Thus, the use of these techniques can help to detect and even quantify the contaminated part of the PP recycling chain with biopolymers, being very important for the PP-based materials final applications and/or to further eliminate the presence of impurities in recycled PP.

## Figures and Tables

**Table 1:** Samples acronym.

Sample	PP1B (wt.%)	PLA (wt.%)	PHB (wt.%)	TPS (wt.%)
PP	100.0	-	-	-
PP-2.5%PLA	97.5	2.5	-	-
PP-5%PLA	95.0	5.0	-	-
PP-7.5%PLA	92.5	7.5	-	-
PP-10%PLA	90.0	10.0	-	-
PP-15%PLA	85.0	15.0	-	-
PP-2.5%PHB	97.5	-	2.5	-
PP-5%PHB	95.0	-	5.0	-
PP-7.5%PHB	92.5	-	7.5	-
PP-10%PHB	90.0	-	10.0	-
PP-15%PHB	85.0	-	15.0	-
PP-2.5%TPS	97.5	-	-	2.5
PP-5%TPS	95.0	-	-	5.0
PP-2.5%TPS	92.5	-	-	7.5
PP-10%TPS	90.0	-	-	10.0
PP-15%TPS	85.0	-	-	15.0

**Table 2:** Values of the solubility parameters calculated from the present constants.

Polymer	Structure	Calculated Small *δ*_(cal)_(MPa^1/2^)	Available Date *δ*_(cal)_(MPa^1/2^) [42]
PP	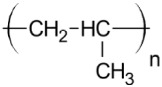	16.4	15.5–17.5
PLA	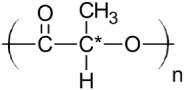	19.5	19.2–21.1
TPS	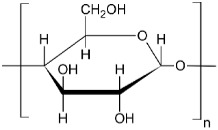	8.4	-
PHB	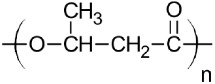	21.4	19.2

**Table 3:** Small’s molar attraction constants for some functional groups [38].

Group	F ((cal·cm^−3^)^1/2^ mol^−1^)
–CH_3_	214
–CH_2_–	133
–CH<	28
>C<	-93
–OH	83
–O–	70
–H (variable)	80–100
>C=O	275

**Table 4:** DSC results of PP blends with biodegradable polymers.

Sample	Tc (°C)	ΔH_c_ (J·g^−1^)	T_m_ (°C)	ΔH_m_ (J·g^−1^)
PP	124.5	85.5	164.1	66.0
PP-5%TPS	121.1	89.0	164.0	66.4
PP-10%TPS	120.5	77.4	164.0	57.3
PP-15%TPS	120.8	67.5	163.7	49.3
PP-5%PHB	120.9	80.5	163.9	62.8
PP-10%PHB	120.8	71.0	164.3	65.1
PP-15%PHB	120.8	69.2	165.1	58.1
PP-5%PLA	121.6	80.2	163.4	56.9
PP-10%PLA	121.5	80.4	163.6	62.0
PP-15%PLA	120.9	70.3	165.1	53.5

**Table 5:** Elongation results of PP blends with biodegradable polymers.

Sample	Elongation at Break (%)	Standard Desviation (%)
PP	4.87	0.61
PP-5%PLA	4.11	1.84
PP-15%PLA	3.29	1.26
PP-5%PHB	3.09	1.39
PP-15%PHB	3.54	1.78
PP-5%TPS	2.26	0.33
PP-15%TPS	5.68	1.5
